# A Protection-Motivation Perspective to Explain Intention to Use and Continue to Use Mobile Warning Systems

**DOI:** 10.1007/s12599-021-00704-0

**Published:** 2021-06-16

**Authors:** Diana Fischer-Preßler, Dario Bonaretti, Kai Fischbach

**Affiliations:** 1 Chair in Information Systems and Social Networks, University of Bamberg, An der Weberei 5, 96047 Bamberg, Germany; 2 Department of Decision Sciences, H. W. Huizenga College of Business and Entrepreneurship, Nova Southeastern University, 3301 College Ave, Fort Lauderdale, FL 33314 USA

**Keywords:** Protection motivation theory, Emergency notification, Disaster, Warning app, Use intention, Continuance, Multi-group analysis

## Abstract

Mobile emergency warning apps are essential for effective emergency communication – of course, provided the population intends to use them. Drawing on protection motivation theory, the study validated a psychometric model to explain what motivates individuals to install a warning app for the first time and to keep using it over time. Multi-group covariance-based structural equation modeling was used to model the answers to a survey that measured the drivers of intention to begin using or intention to continue using a warning app. The model shows that, for both non-users and users, trust, social influence, and response efficacy positively and maladaptive rewards negatively affect intention to use and intention to continue use warning apps. However, perceived vulnerability influences only intention to use, whereas response cost and self-efficacy affect continued use intention. Hence, this study enhances the theoretical understanding of technology-enabled protection behaviors and provides practitioners with a list of factors to consider for pushing the adoption and continued use of emergency warning applications.

**Supplementary Information:**

The online version contains supplementary material available at 10.1007/s12599-021-00704-0.

## Introduction

The popularization of smartphones and the ubiquity of the internet has turned mobile-enabled emergency communication into a crucial asset for alerting populations in cases of emergency. Around 2010, public authorities began to distribute verified warning messages during emergencies using mobile *warning software applications* (hereafter “warning apps”), which are software applications that run on devices (e.g., smartphones, smartwatches) for one-way warning communication from authorities to relevant publics (Fischer-Preßler et al. 2020; Tan et al. 2017). The use of these warning apps increases the resilience of the population to crises, as people can take informed actions upon receiving an alert.

Historically, different channels have been used to transmit emergency warnings, such as radio, television, newspapers, and sirens (Botterell and Addams-Moring 2007; Mayhorn et al. 2006). The widespread use of mobile devices and lightweight IT (Bygstad 2017) offered a new channel for precise, immediate distribution of emergency-related information (Leelawat et al. 2013; Meissen et al. 2014; Valtonen et al. 2004). Examples include warning apps such as the FEMA app, NOAA Weather Alerts, and KATWARN. In 2020, a new form of warning app – contact tracing apps – came into widespread use to help cope with Covid-19 by alerting users who have come into close contact with infected people. Their effectiveness depends on attaining a critical number of users (Trang et al. 2020). In addition, the higher the penetration rate of warning apps among the population, the more effective the warning channel. The penetration level of these apps, as well as maintaining a stabile user-base over time, depends on people’s intention to use them and intention to continue doing so over time.

Public authorities seeking to promote and leverage the use of warning apps must understand what drives their use. That is paramount for developing strategies to promote them among the population and effectively alert people in emergency situations. Few studies, however, have investigated people’s intention to use an emergency app (for a review see Tan et al. 2017). There is research on the use of mobile apps other than those for emergency communication (e.g., social media, fitness apps), but scholars have argued it may suffer generalizability issues when applied to warning app use. Thus, researchers have advocated for more context-specific studies of app use (Hoehle and Venkatesh 2015).

Warning apps and their use differ from other apps for at least two reasons. First, they run in the background, and interaction with them, solicited by a warning, is infrequent because emergencies are rare events. Second, the decision to install and use a warning app constitutes protective behavior against the negative effects of potential emergencies. Unlike hedonic apps (van der Heijden 2004) such as social media, the design objective of warning apps is not to encourage prolonged use based on providing a user with, say, enjoyable experiences, but rather to guide user behavior during emergencies. The use of a warning app reflects a protective behavior because it put users in the position to receive timely warnings and cope with potential threats.

Information systems (IS) researchers have studied technology-enabled protective behavior as manifested in two main dependent variables. The first is compliance: following a warning message’s recommendations to respond to an emergency. For example, researchers have examined the antecedents of an individual’s intention to comply by focusing on SMS warning of US campus warning systems (e.g., Han et al. 2015). The second is use of a warning system, which expresses a protective behavior in itself. Specifically, IS security research has focused on intention to use security IS as an expression of technology-enabled protective behavior (e.g., Boss et al. 2015; Lee and Larsen 2009). In addition, research into campus emergencies has focused on the use of SMS-based warnings (Lee et al. 2013). Thus far, the “compliance” and “use” perspectives have not informed each other and have not been combined to seek a conclusive explanation for the intention to protect oneself by using or continuing to use a warning app.

Further, research has focused on examining the drivers of initial use intention while overlooking the drivers of *continued* use intention (e.g., Ada et al. 2016; Lee et al. 2013). Keeping a population highly vigilant, however, also relies on *continued* use intention. For instance, the success of Covid-19 tracing apps depends on the number of users who continue to use the apps over time as much as it does on those who begin using them. As countries recover from the pandemic and the perceived risk of contracting the virus decreases, however, more individuals may tend to discontinue tracing app use. This research informs strategies to foster both use and continued use of warning apps by identifying the determinants of use intention and continued use intention. Our research question is:

What drivers and impediments explain non-users’ intention to use a warning app and users’ continued use intention?

To answer this question, our research model synthesizes research on the use of mobile emergency warning systems and protection-motivated use of technology. We draw on prior research on protection motivation theory (PMT) (Rogers 1975, 1983) because it has been applied successfully to understand a diverse array of IT-related protective actions (Lee and Larsen 2009). We complement the model by incorporating relevant factors in the context of mobile-enabled emergency warning to determine the most important factors of warning app use (e.g., Han et al. 2015). In particular, our analysis focuses on the drivers of non-users’ warning app use intention and users’ continued warning app use intention.

From a theory development standpoint, we advance the understanding of PMT by contextualizing it to mobile-enabled emergency communication to explain non-users’ and users’ warning app use intentions. In doing so, we indicate differences and similarities between non-users and users and identify major drivers of use intention within both groups. Our findings provide public authorities and relief organizations with a theoretical framework for promoting warning apps among the population.

## Literature Review

### Emergency Warning and Warning Apps

Warning apps are software applications running on mobile devices (e.g., smartphones, smartwatches) that disseminate warnings to a threatened population (Fischer-Preßler et al. 2020). We define a *warning* as a safety communication to inform a population about a threat from an imminent or ongoing emergency. Its goal is to instruct recipients regarding how to respond to the emergency to avoid or minimize undesirable consequences. In particular, in addition to descriptions of emergency type (e.g., flood, terrorist attack), warning messages typically include recommended actions to help those affected respond to the emergency (Fischer-Preßler et al. 2020).

Warning through mobile applications has gained popularity as the use of mobile devices has grown (Reuter et al. 2017). There are at least three peculiarities of warning apps that distinguish them from other warning channels. First, warning apps put recipients in a state of alert through push notification and a digital representation of the emergency. Compared to other channels such as TV and radio, warning apps are designed to activate the user even when the apps are only running in the background. Compared to sirens, which can only warn people about the existence of a threat, warning apps can communicate much richer information about an emergency. Second, warning apps are considered a source of trustworthy information, since they are managed by public authorities; for instance, users know that the U.S. Federal Emergency Management Agency (FEMA) is the source for the FEMA warning app (Tan et al. 2017). This distinguishes warning apps from other channels through which unverified emergency-related information may be diffused, such as in social media. Third, users can set apps so they are warned only about events most relevant to them, such as certain types of events or only those in predefined locations.

### Warning App Use Intention and Continued Use Intention

IS scholars are extremely prolific in research on *use* intention and *continued use* intention (for reviews, see, for instance, Franque et al. 2020; Venkatesh et al. 2016). Mere use intention refers to the intention of using a system for the first time. Continued use intention, instead, refers to current users using the system into the future (Bhattacherjee and Lin 2015). Continued use is seen as an intention resulting from a rational decision to use the technology based on beliefs about, expectations of, or experience with that technology (Ortiz de Guina and Markus 2009). Thus, throughout the manuscript, we refer to “use intention” for non-users and “continued use intention” for current users.

Research in the mobile-enabled warning context has investigated factors that motivate students to subscribe to receiving emergency SMS (Ada et al. 2016; Bonaretti and Fischer-Preßler 2021), adopt social media services for emergency warnings (Lee et al. 2013), and comply with emergency warning systems (Han et al. 2015), but only in the context of campus communities. However, the unique cultural and institutional conditions of student populations render it difficult to generalize those findings. For example, students’ sense of attachment to their campus communities may pressure them to comply with university guidelines. This limitation calls for additional evidence to test whether results from studies on use intention of campus warning systems also replicate in the warning app context. In addition, these studies focused only on use intention and did not investigate antecedents of continued use intention.

In terms of continued use, researchers have applied a wide array of theories to explain continued use intention outside the emergency context, including expectation-confirmation (e.g., Bhattacherjee 2001; Chen et al. 2012), acceptance (e.g., Baptista and Oliveira 2015; Venkatesh et al. 2003), and social capital (e.g., Chang and Zhu 2012; Franque et al. 2020). However, as we argue, theories applied to understand continued use of hedonic systems do not suit the emergency context, in which most interaction with the app is prompted by occasional emergency notifications. In particular, explaining continued use with theories that consider drivers such as satisfaction, hedonic value, habit, flow, or perceived enjoyment (Franque et al. 2020), while appropriate for hedonic systems (Warkentin et al. 2016) such as social media apps (Chang and Zhu 2012), is less appropriate for emergency warning systems. For instance, while theories on IS continuance stress the role of satisfaction in the sense of a pleasurable user experience for the continuance of use intention (Bhattacherjee and Premkumar 2004; Shaikh and Karjaluoto 2015), this determinant is less relevant with respect to protective behavior. IS use in the context of security behavior is not aimed at personal satisfaction, as it is in hedonic systems (Warkentin et al. 2016).

With context being that crucial, we considered literature that studied use intention and continued use intention as manifestations of protection behavior for the development of our research model. Our particular attention was on studies of use and continued use in the context of IS security, because using security systems is a form of protective behavior (Vedadi and Warkentin 2020; Warkentin et al. 2016). Specifically, we focused on research that draws on the theory of protection motivation introduced in the next section.

### Protection Motivation Theory

Our perspective to study mobile-enabled protective behavior draws on protection motivation theory (PMT), originally developed to explain protective behavior in health and social psychology (Rogers 1975, 1983). Rooted in expectancy-value theory, PMT explains the social and cognitive processes underlying protective behaviors. The theory hinges on the notion that the decision to counteract a threatening event is a function of *threat* and *coping appraisal processes*. Threat appraisal refers to the perceived likelihood that the event will indeed occur and have negative consequences. The perceived level of the threat depends on an individual’s assessment; it is based on direct (e.g., loss of property, evacuation) and indirect (e.g., reading information, word of mouth) experiences with emergency events (Martin et al. 2007). Coping appraisal refers to the expected efficacy of counteracting a threatening event. For protection motivation to occur, the perceived efficacy of a protective behavior must outweigh the costs of the counteractive behavior (Maddux and Rogers 1983; Rogers 1975, 1983). Environmental sources of information such as verbal persuasion or observational learning may initiate these processes, as could intrapersonal sources such as experience (Milne et al. 2000). Counteraction instantiates into an adaptive response (e.g., complying with a recommendation) rather than a maladaptive (i.e., non-adaptive) one. In particular, if individuals conclude that a threat will affect them, they will be more motivated to protect themselves and will thus initiate, continue, or cease a certain self-protective behavior (Rogers 1975).

We chose PMT because the focus of this study is at the intersection of the domains where PMT has proven valid. Scholars have applied PMT to study protective behaviors in different domains such as health promotion and disease prevention (e.g., Floyd et al. 2000; Milne et al. 2000), environmental concerns (Bockarjova and Steg 2014), organizational commitment (Posey et al. 2016), automobile accident prevention (Glendon and Walker 2013) and other areas of interests beyond health-related behavior (e.g., Bubeck et al. 2012; Chakraborty et al. 2014). In IS research, PMT has been applied mainly in security IS (Menard et al. 2017 and, for a review, see Boss et al. 2015), such as home computer security behavior (Anderson and Agarwal 2010), the use of an email authentication service (Herath et al. 2014), adoption of security technologies (Lee and Larsen 2009) or anti-virus software (Lee et al. 2008), and strong passwords (Zhang and McDowell 2009). The commonality among these studies is the attempt to use PMT to explain the intention to initiate, maintain, or cease a certain protective behavior.

This field of research is relevant to emergency communication because warning apps are intended to activate protective behaviors. Using a warning app is per se a protection motivation manifestation because a warning app bears a protective function, that is, to warn the user in case of an emergency. In particular, in the context of our research, protection motivation instantiates in the intention to *use* or intention to *continue to use* the warning app.

### Differences Between Non-Users’ and Users’ Protection Motivation

When studying use from a protection-motivation perspective, there are differences between users’ protection-motivation, which requires a decision regarding whether to continue to use, and non-users’ motivation, which requires a decision about whether to initiate use. For instance, while a perceived threat is a relevant factor for continued use intention of IS security systems (Warkentin et al. 2016) as well as for initial use intention (e.g., Boss et al. 2015), perceived response efficacy is significant to motivate initial use (e.g., Boss et al. 2015) but not continued use (Warkentin et al. 2016). We speak cautiously of “potential” differences because groups of non-users and users have not been directly juxtaposed in the IS security literature.

In prior research, the differences between non-users and users has been studied for mobile-enabled SMS e-government services, which have traits in common with warning apps (e.g., the source is a public authority, use is non-hedonic). Compared to non-users, users were indifferent to perceived costs and risks of using the system, behavioral control, and convenience (Susanto and Goodwin 2013). One explanation for this difference is that users, once they adopt a system, are willing to incur costs (e.g., of receiving SMS notification), become more self-confident of their ability to use the system, and are more aware of whether using the system helps fulfill an intended purpose. Thus, explanatory variables of continued intention to use do not fully generalize to explain non-users’ intention to begin to use a system. However, this difference among non-users and users has not been investigated in the context of warning apps.

## Model and Hypotheses Development

As explained above, PMT explains adaptive behaviors from an expectancy-value perspective. The decision to undertake an adaptive behavior entails two appraisal processes: threat and coping appraisals. First, individuals facing physical threats assess if and how these threats will affect them and whether they outweigh maladaptive rewards (threat appraisal). In particular, PMT postulates that threat appraisal is more likely to prompt an adaptive behavior when perceived severity and vulnerability outweigh the rewards that may result from persisting in the current state. In our case, public authorities may recommend using a warning app to receive timely alerts and thus minimize losses, but the risk may be perceived as trivial. Thus, following PMT, individuals may conclude that an emergency is unlikely to affect them and not use the app. Conversely, threats perceived to be high may lead individuals to use the app. Thus, our research model incorporates the three PMT dimensions of threat appraisal: *perceived severity*, *perceived vulnerability*, and *maladaptive rewards* (Boss et al. 2015).

Second, individuals compound the benefits of performing the protective behavior against its costs (coping appraisal). Then, an adaptive behavior will occur only when the benefits from complying outweigh associated costs. Specifically, coping appraisal is more likely to prompt adaptive behavior when response efficacy and self-efficacy are greater than the detriment from using a warning app (perceived cost). In the context of this research, individuals may evaluate using a warning app as being an efficient means (e.g., to be timely alerted) coping effectively with the emergency against the efforts associated with using it. Hence, we include the three dimensions of coping appraisal: *response efficacy*, *self-efficacy*, and *response cost* (Boss et al. 2015).

The emergency management context offers a unique scenario in which *system use intention* and *continued use intention* can be interpreted as *protection motivation*, and determinants of IT-elicited protective behaviors can be used to explain those intentions. Following this reasoning, we posit that studies on compliance intention with warning messages (e.g., “shelter in place”) (Han et al. 2015) and adoption of warning apps (Appleby‐Arnold et al. 2019) can also be interpreted from a PMT perspective, that is, their outcome variables – whether use of a warning system or compliance intention with its warning messages – correspond to protection motivation (Boss et al. 2015). While PMT characterizes protective behavior as the result of an expectancy-value analysis, studies on warning messages argue that receivers’ protection behavior is instead a function of social and institutional contexts. Drawing on Etzioni’s compliance theory, Han et al. (2015) included social influence to account for the peer pressure that influences people’s protection motivation. Furthermore, they added information quality trust to capture the effect of warning message credibility in eliciting intention to act upon the information in the warning message. Both *information quality trust* and *social influence* emerged as dominant determinants in the context of campus emergency communication (Ada et al. 2016; Han et al. 2015). Thus, adding information quality trust and social influence to PMT seems critical for a more complete explanation of protection motivation in the emergency warning context.

**Fig. 1:**
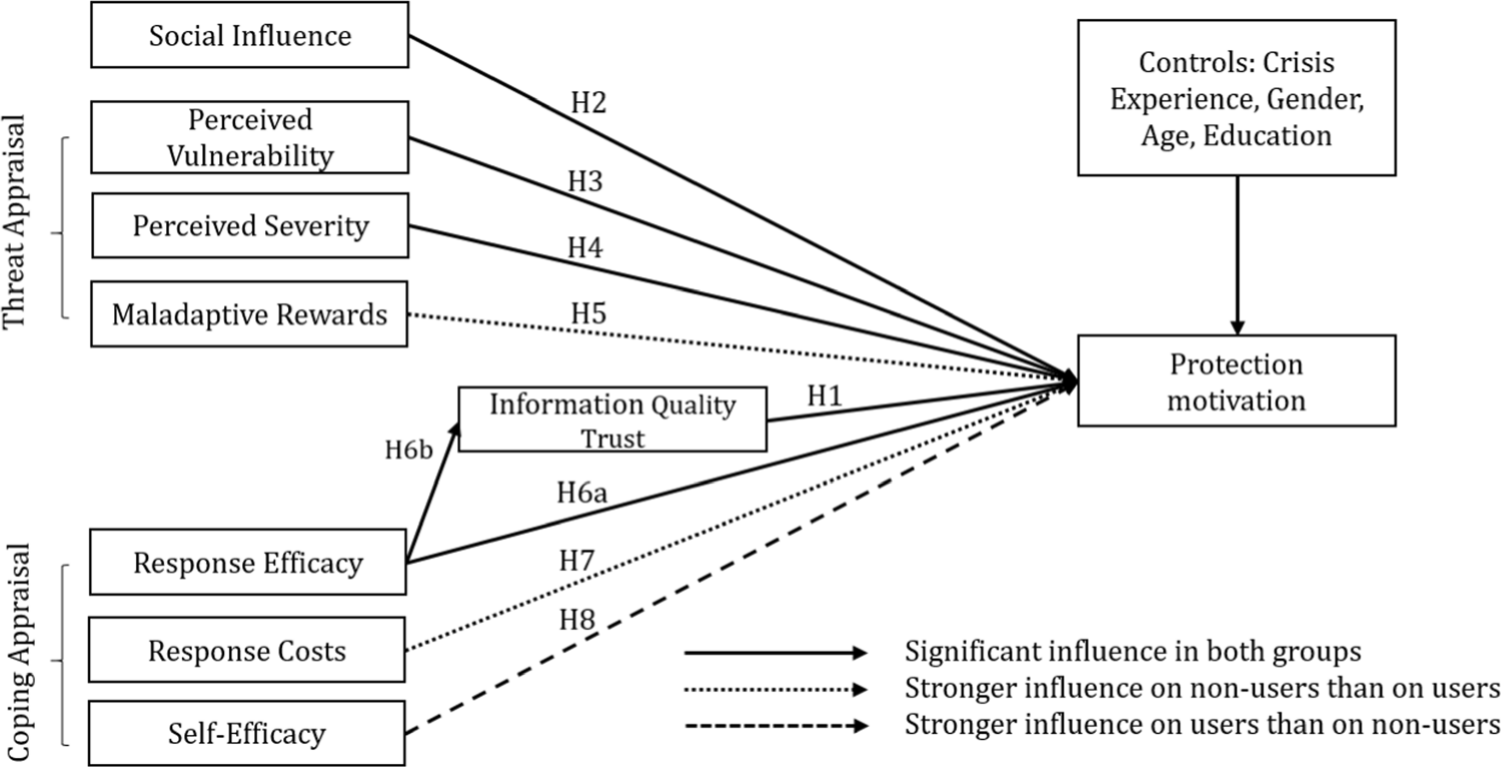
Research model

Figure 1 shows our research model and the proposed hypotheses. The following section describes our hypotheses development.

Information quality trust is a significant determinant of human behavior during emergency events (Han et al. 2015). This construct captures the users’ expectation that the information source (i.e., authorities) will disseminate relevant, actionable, and critical information. Users must perceive warnings from the app as trustworthy for them to be actionable. PMT originally did not explicitly incorporate information quality trust, but it included the source of information as a trigger for protective behavior (Rogers 1983). From an information quality perspective, however, any given information source carries a certain level of trustworthiness on which users base their protection motivation. Therefore, integrating PMT with information quality trust accounts for the trustworthiness of the “source of information” neglected by PMT research to date.

Trust in the information provided, in turn, is critical for successful authorities-to-people communication, because people who trust authorities as credible sources of information are more willing to use or continue to use a warning app (Appleby‐Arnold et al. 2019). A lack of trust in information quality, in contrast, conflates with lack of trust in a given source (Glik 2007) because trust in the information provided depends on trust in the message’s dispatcher – the public authority – as does the acceptance of emergency warnings (Appleby‐Arnold et al. 2019). Trusting the information dispatched is a precondition to following authorities’ suggested protective behavior (Lindell and Perry 2012). This result was corroborated by research on campus warnings that identified trust as a main significant predictor of intention to comply with warning messages during emergencies (Han et al. 2015). Moreover, because using warning systems is in itself a protective behavior, we can infer that trust also positively influences use intention of campus emergency alert systems (Ada et al. 2016) and use of disaster apps (Appleby‐Arnold et al. 2019).

Warnings are useful only if the information is credible, reliable, and actionable. Thus, users’ perception of the trustworthiness of the information is a main reason to continue using the app (Appleby‐Arnold et al. 2019). If the app meets users’ expectations in terms of information quality, higher confidence in the technology and lower uncertainty about the system’s reliability will increase continued use intention. This also conforms with research on public e-services, which identified trust as the main determinant for continued service use (Belanche et al. 2014). We thus hypothesize that trust has a similar positive effect on protection motivation both for non-users and users.

### H1

Information quality trust has an equally positive effect on protection motivation of non-users and users.

Social influence is an individual’s motivation to comply with the expectations of relevant others (Han et al. 2015). It is based on normative beliefs, that is, an individual’s perceived behavioral expectations of relevant others such as family, friends, or supervisors (Venkatesh et al. 2003). Generally, social factors have been found to have a strong influence on security IT adoption and use (e.g., Lee and Larsen 2009). There is also evidence from warning compliance research that people will be more likely to comply if they perceive that a given behavior is expected by their relevant others (Han et al. 2015; Lee et al. 2013). In addition, some people even perceive adopting available warning systems as a civil duty and moral obligation (Appleby‐Arnold et al. 2019). Social influence was also identified as a significant determinant for adoption behavior in PMT research (Lee and Larsen 2009) as well as in PMT research in the context of disaster risk reduction (McCaughey et al. 2017). Thus, prior research supports that social influence may play a critical role in all kinds of protective intentions.

In terms of the effect of social influence on continued use intention, protection motivation-related studies do not provide explanations. Generally, social influence could become less important for IS use over time, as users potentially develop first-hand opinions regarding a given technology’s usefulness (Venkatesh and Davis 2000). However, research on IT continued use intention in various contexts found that social influence not only influences adoption, but also remains a crucial factor influencing users’ continued IT use intention (Franque et al. 2020). In the context of warnings apps, the expectations of key referents such as family and friends could influence similarly both continued use intention and adoption intention, as warning apps concern a user’s personal safety. Important others may thus expect and encourage app use, since it promotes the safety of a loved one. For non-users, prior research found social influence has a significant influence on protection motivation (Han et al. 2015). Hence, we posit that social influence will positively affect protection motivation alike for both groups.

### H2

Social influence has an equally positive effect on protection motivation of non-users and users.

An emergency can cause both physical (e.g., property damage, casualties) and social disruption (Lindell 2013). Thus, the decision to adopt and use a warning app can be explained as a function of individuals’ assessments of their perceived exposure to such risks. In particular, individual *perceived vulnerability* is how likely an individual expects to experience an emergency first-hand. People’s perception that an emergency will affect them positively influences protection motivation. *Perceived severity*, in contrast, is how harmful one expects the consequences of an emergency to be on things that matters to the individual (e.g., personal health and security). According to PMT, an individual’s *perceived vulnerability* to a threat, and *perception* of its *severity,* increase protection motivation (Boss et al. 2015). These hypothesized effects align with research on campus emergencies, which confirmed that perceived probability and severity of an emergency positively affect compliance in cases of active shooter events and building-related incidents (Han et al. 2015).

In terms of continued use intention in IS security, Warkentin et al. (2016) found significant influence of perceived threat severity and vulnerability on continuance behavior of an anti-malware software. When the system is a warning app, protection motivation relates to the perception that the app will help protect against the effects of an emergency by warning in a timely manner and suggesting appropriate safety measures. Users must perceive that somewhat severe emergencies could happen for which they need to be warned about – otherwise mass warnings would be unnecessary and warning apps would be useless. So only a sustained level of threat perception will lead to protection motivation. In the context of our study, that means non-users as well as users are more likely to protect themselves by using or continuing to use a warning app if they perceive that an emergency event is likely and may be severe.

### H3

Perceived vulnerability has an equally positive effect on protection motivation of non-users and users.

### H4

Perceived severity has an equally positive effect on protection motivation of non-users and users.

*Maladaptive rewards* are opportunities associated with not using a warning app, such as saving battery charge or storage space on a smartphone. If these rewards outweigh a perceived threat, individuals will opt not to protect themselves against that threat (Boss et al. 2015). While studies of information security often omit maladaptive rewards, Boss et al. (2015) demonstrated that maladaptive rewards have a positive effect on anti-malware software use intention and stressed the importance of the construct for PMT application. Following this argument, if people perceive that the app requires too much system capacity compared to the benefits of using it, they are less likely to use the app. However, once users become familiar with it, they may feel that the app does not typically require much system capacity after all. Thus, maladaptive rewards do less to discourage continued use intention of the system. Non-users, in contrast, might consider smartphone performance issues to be more relevant in their decision to begin using the app. Thus, we claim that the negative relationship between maladaptive rewards and protection motivation is stronger for non-users than for users.

### H5

The negative effect of maladaptive rewards on protection motivation will be stronger for non-users than for users.

According to PMT, *response efficacy* influences protection motivation behavior (Maddux and Rogers 1983; Rogers 1983). Perceived response efficacy is the belief that the adaptive response (i.e., use of a warning app) will be effective in protecting someone (Boss et al. 2015). Pura (2005) found that people are more likely to use a location-based mobile service if it creates new value in the context of its use. A protective behavior seeks to provide an acceptable level of safety against threats (Warkentin et al. 2016). Therefore, people must perceive the app as useful for protecting them during emergency events (i.e., that they transmit warnings in a timely manner without requiring further action on the smartphone users’ part).

IS security research investigating the effect of response efficacy on continued use intention has conflicting results. While Warkentin et al. (2016) found no significant effect on this relationship in the context of anti-malware software, Vedadi and Warkentin (2020) found modified response efficacy to be a main driver of continuance intention in the context of password protection. For initial adopters, the positive effect of response efficacy instead was confirmed widely (e.g., Boss et al. 2015). Ultimately, whether response efficacy influences protection motivation of non-users and users alike remains unclear. In the context of emergencies, individuals will deem protective measures to be helpful if they perceive them as effective (Grothmann and Reusswig 2006). A system that proves inefficient in protecting one from a given threat is not likely to be used in the long term. Thus, we claim that high levels of perceived response efficacy will positively affect protection motivation among both non-users and users.

### H6a

Response efficacy has an equally positive effect on protection motivation of non-users and users.

In addition, research on trust and the perceived effectiveness of authorities indicates that people trust them when they perceive public authorities and emergency responders to be acting effectively. Thus, people’s perception that authorities distribute information effectively via the app positively affects trust (Appleby‐Arnold et al. 2019). Trust builds on success, such as individuals recalling that authorities warned the population in a timely manner during a prior emergency. Trust may decrease, even for non-users, if people learn about failures to issue timely warnings. Thus, we hypothesize:

### H6b

Information quality trust partially mediates the relationship between perceived response efficacy and protection motivation for both non-users and users.

Use of a warning app requires that people take some action, such as installing the app, changing phone settings, and/or updating operating system to versions needed to support the app. Thus, there are *response costs* associated with using a warning app specific to the time and effort required to carry out a safety behavior (Boss et al. 2015). As the cost or effort required to perform the actions increase, intention to carry out the behavior decreases. If people perceive costs related to warning app use as too high, they are less likely to carry out the behavior. This is in line with prior research that found perceived costs to have a negative influence on attitudes toward mobile services (Susanto and Goodwin 2013). That said, warning app use is not very time consuming and does not require much effort beyond installation, initial set up, and updating. Further, they primarily run in the background and use is infrequent. Users familiar with an app might be more willing to incur in those costs than non-users. Thus, costs could appear less onerous to those choosing to continue using the system than for non-users who have yet to familiarize with the system. Thus, we hypothesize:

### H7

The negative effect of perceived response costs on protection motivation will be stronger for non-users than for users.

Self-efficacy (H8) is the level of confidence in people’s ability to use warning apps to protect themselves (Boss et al. 2015). It has been found to have a strong effect on the intention to take protective actions (Milne et al. 2000). If individuals are confident in their ability to effectively carry out protective actions and those actions are not difficult, they are more likely to perform the behavior. The effect of self-confidence has been confirmed in the IS security research context in driving decisions such as the adoption of anti-malware software (e.g., Boss et al. 2015). Transferring these findings to the warning app context, we can say that individuals who are more confident that they can easily use an app, are more willing to do so because they do not perceive barriers to use.

In IS security behavior, self-efficacy has been found to have a positive effect on IS continuance (Warkentin et al. 2016) and initial adoption intention (Boss et al. 2015). However, its effect has never been investigated simultaneously among non-users and users. We hypothesize that the effect is stronger for users than for non-users, because users who fully adopt a system become more self-confident in their ability to use the app over time. Hence, we hypothesize:

### H8

The positive effect of perceived self-efficacy on protection motivation will be stronger for users than for non-users.

Furthermore, prior studies have often considered experience with a crisis to have a powerful effect on the recognition of a threat and to be an important factor influencing protective behavior (Bubeck et al. 2012; Thieken et al. 2007). Generally, people who experienced emergencies before will perceive such events as happening more frequently than they actually do, and see themselves as potential future victims (Weinstein 1989). Consequently, they may be more likely to engage in protective behavior. To control for potential differences in the threat appraisal process between people with and without such experiences, we added a control variable for prior emergency experience. Finally, in line with prior research, we controlled for age (Appleby‐Arnold et al. 2019), gender, and education (Anderson and Agarwal 2010).

## Methodology

### Data Collection and Sample Description

We used survey data to test the relationships in our research model. Following Anderson and Agarwal (2010), we developed a survey that provides contextual information to ensure respondents complete it while thinking about recent emergency events and the use of a warning app to respond to an emergency threat. Since we conducted our study in Germany, we included several crisis events that have occurred there since 2016, such as the flash flooding in May and June 2016, the shooting rampage at a Munich mall, and a chemical accident in Oberhausen in 2017. Further, we explicitly defined the term “warning app” to ensure that respondents would have a common understanding of the subject. In the introduction, the questionnaire explained the features and properties of warning apps and provided examples of the two most popular German warning apps: NINA and KATWARN. Thus, respondents who were not previously aware of warning apps were provided with the information necessary to understand the intended use of such apps.

The survey was distributed in digital form through a URL posted in different German Facebook groups, such as those for new residents in a city or those for scientific studies. Our three reasons for administering the questionnaire via Facebook outweigh the concerns of self-selection bias in our sampling method. First, Facebook facilitates reaching out to a relevant subpopulation of those who own smartphones, which is a necessary condition to be (or become) a warning app user. In 2017, some 21 percent of the German population used Facebook on a daily basis; of that group, 72 percent accessed it primarily via their smartphones (Frees and Koch 2018). Second, we wanted to control for the differences between people who have and have not experienced emergency events. Third, because of the sensitive nature of the topic, we wanted to guarantee respondent anonymity (Kosinski et al. 2015), a necessary element in eliciting honest responses to questions about emergency perception and experience. Since previous research has shown that Web administration of questionnaires mitigates social desirability bias (Kreuter et al. 2009), administering the questionnaire via Facebook was our best choice. While recruiting respondents only through Facebook and making participation voluntary could cause self-selection bias and limit the generalizability of our results, we control for biases in the Facebook user population by adding control variables that prior literature considered relevant.

A total of 459 individuals took part in the survey from March to June 2017: 178 women, 272 men, and 5 unspecified. With respect to education level, the sample includes respondents with a Certificate of Secondary Education (4.4%), General Certificate of Secondary Education (22.9%), Higher Education Entrance Qualification (31.4%), University Degree (38.8%), and not specified (2.2%). The mean age of respondents is 33.46 years (*SD* = 11.939).

### Measurement Model

#### Operationalization and Development of the Measurement Scales

We developed the initial set of question items from existing scales. The items for the latent constructs were then contextualized to our domain to enhance validity and reliability. All the reflective indicators for our constructs were collected on a 7-point Likert-scale (1 = strongly disagree; 7 = strongly agree). Moreover, we collected control variables for gender (male/female), experience with emergency events (yes/no), age, and level of education (categorical). We further asked whether people have already used NINA, KATWARN, or similar warning apps. The items were pretested with a convenience sample of postgraduate students. We collected comments regarding, among others, the clarity and structure of the items, and we measured the time required to answer the entire questionnaire. We further assessed the internal consistency of the measurement scales by means of coefficient alpha estimates. Based on the results, we revised the questionnaire and modified several items for the final study. Table B1 in the Appendix (available online via http://link.springer.com) is an overview of the final measurement scales.

#### Data Preparation

We excluded three respondents of the 459 responses because they presented a large amount of missing data (> 41.5%). In the remaining sample (N_non-users_ = 226; N_users_ = 230), there were only 18 respondents with missing data; most of them answered all but one of the items. We observed no discernible pattern in the missing values. Hence, we could estimate our model with full information maximum likelihood (FIML) (Arbuckle 1996) as long as the other assumptions of covariance-based structural equation modeling were fulfilled.

#### Analysis Approach

To test hypotheses, we estimated the path model using a covariance-based approach (SEM) rather than a variance-based approach (PLS). We did so for several reasons: (1) we wanted to avoid biased path modeling parameter estimates; (2) we intended to model the measurement error variance and rely on a factor analytic measurement model; (3) we followed a confirmatory approach with our analyses; (4) we did not use any formative indicators; (5) we had a large sample size; and (6) we did not have any problems ensuring convergence (Gefen et al. 2011; Reinartz et al. 2009).

#### Measurement Model Analysis

We assessed the psychometric properties of the scales through a confirmatory factor analysis (CFA) on each group. A battery of fit indices (Hu and Bentler 1999) calculated with AMOS 25.0.0, indicated a good model fit for non-users and users (see Model 2 and 3 in Table 1). In the next step, we tested the CFA model on the two groups simultaneously. The results showed a good fit (see Model 4 in Table 1) indicating that the measurement properties of the model fit the two groups well. Concerning convergent validity, the factor loadings of all indicators for the latent constructs were greater than the 0.7 benchmark and highly significant (*p* < 0.001) (see Tables in the Online Appendix). Furthermore, Cronbach’s Alpha was greater than 0.90 for all constructs. In addition, the construct reliabilities exceeded 0.6 (Bagozzi and Yi 1988), and the average variances extracted (AVEs) of all constructs exceeded 0.5 (Fornell and Larcker 1987). Since the square root of the AVE for each construct was greater than the correlation of each construct with all other constructs, we also found evidence for discriminant validity (Fornell and Larcker 1987). Furthermore, we tested for common method bias (CMV) using a single unmeasured latent method factor (Podsakoff et al. 2003). The difference in goodness of fit between the CFA models with and without the single method factor was not significant (χ^2^/df = 0/1), which suggested that CMV is not a threat.

**Table 1:** Tested measurement models

	χ^2^	Df	Sig	CMIN/DF	CFI	IFI	RMSEA	NFI	TLI
Model 1: Baseline measurement	612.509	314	0.000	1.951	0.979	0.979	0.046	0.958	0.973
Model 2: Measurement model (users)	502.007	314	0.000	1.599	0.962	0.963	0.051	0.907	0.951
Model 3: Measurement model (non-users)	614.509	314	0.000	1.051	0.979	0.946	0.062	0.958	0.973
Model 4: Simultaneous measurement model	1038.338	628	0.000	1.653	0.967	0.968	0.038	0.922	0.958
Model 5: Constraint measurement model (factor loadings restricted)	1109.274	647	0.000	1.714	0.963	0.964	0.040	0.917	0.954

### Structural Model

#### Structural Model Results

To test the hypotheses that related to the structural relationship, we employed a multi-group approach using structural equation modeling analysis (see Table 2). Prior to the multi-group SEM analysis (model 10), the model fit to the data from each group was tested separately. Fit indices indicated a good model fit for non-users and users (see Model 8 and 9).

**Table 2:** Tested structural models

	χ^2^	Df	Sig	CMIN/DF	CFI	IFI	RMSEA	NFI	TLI
Model 7: Baseline structural	783.303	399	0.000	1.963	0.973	0.973	0.046	0.947	0.964
Model 8: Structural model (users)	633.706	399	0.000	1.588	0.954	0.955	0.051	0.887	0.939
Model 9: Structural model (non-users)	681.664	399	0.000	1.708	0.963	0.964	0.056	0.917	0.951
Model 10: Simultaneous structural model	1315.372	798	0.000	1.648	0.959	0.960	0.038	0.905	0.946
Model 11: Constrained simultaneous model	1364.152	828	0.000	1.648	0.958	0.959	0.038	0.901	0.946

Having established that the models were a good fit to the observed data of both groups, we examined the model fit to the pooled data across the two groups. The structural model (model 10) explains 69 percent of the variance of warning app use intention and 39 percent of trust, whereas the second model explains 45 percent of the variance of continued warning app use intention and 31 percent of trust. For readability, Table 3 indicates the coefficients and significance levels of our hypothesis testing.

**Table 3:** Group-wise hypothesis testing using standardized beta coefficients and results of the x^2^ difference test

Group effect	Paths	Non-users	Users	Results of the *x*^*2*^ difference test	
		*b (t-values)*	*b (t-values)*	CMIN *(p values)*	Conclusion
Same impact across groups	H1: Information quality trust → Protection motivation	0.128* *(2.414)*	0.192** *(2.725)*	0.000 *(0.989)*	Supported
	H2: Social influence → Protection motivation	0.290*** *(4.688)*	0.280*** *(3.393)*	0.981 *(0.322)*	Supported
	H3: Perceived vulnerability → Protection motivation	0.209*** *(3.795)*	0.155 *(1.730)*	2.891 *(0.089)*	Not supported
	H4: Perceived severity → Protection motivation	− 0.083 *(*− *1.593)*	0.088 *(1.335)*	4.310 *(0.038)*	Not supported
	H6a: Perceived response efficacy → Protection motivation H6b: Perceived response efficacy → Trust	0.280*** *(3.641)* 0.613*** *(10.228)*	0.183^†^ *(1.917)* 0.551*** *(7.757)*	0.648 *(0.421)* 0.876 *(0.349)*	Supported Supported
Stronger influence on non-users	H5: Maladaptive rewards → Protection motivation	− 0.145** *(*− *2.607)*	− 0.249*** *(*− *3.439)*	0.594 *(0.441)*	Not supported
	H7: Response cost → Protection motivation	− 0.115 *(*− *1.411)*	− 0.155* *(*− *1.999)*	0.552 *(0.458)*	Not supported
Stronger influence on users	H8: Self-efficacy → Protection motivation	− 0.050 *(*− *0.973)*	− 0.283*** *(*− *3.514)*	3.657 *(0.056)*	Not supported
Control variables	Gender → Protection motivation Education → Protection motivation Age → Protection motivation Emergency experience → Protection motivation Emergency experience → Trust	− 0.044 − 0.061 − 0.067 0.054 0.101	− 0.007 0.032 − 0.059 − 0.131* 0.077		

R^2^ = total explained variance; R^2^ (use intention) = 0.69; R^2^ (continued use intention) = 0.45

^†^*p* ≤ 0.10; *p ≤ 0.05; **p ≤ 0.01; ***p ≤ 0.001

#### Multi-group Analysis

Testing for differences between users and non-users requires to assess whether the measurement model fits equally well between the two groups. Thus, we tested measurement model invariance by conducting a multi-group comparison in Amos between users and non-users. The model is invariant when the path coefficients and means are the same, regardless of whether the model is fitted using the subsample of non-users and users separately. However, Byrne (2004) noted that complete invariance is often rejected – and our model also failed to meet complete invariance. To address that, she proposed a set of tests to claim certain levels of invariance: configural, metric (or “weak”), and full. The level of variance determines the kind of conclusions that can be drawn. Based on Byrne (2004) categorization, our model showed configural invariance, as the model fit of models 4, 2, and 3 are good. We further tested for weak invariance by constraining factor loadings (measurement weights) of the indicators to be equal among both groups and comparing it to the simultaneous measurement model. That is a necessary condition for comparing the relations between constructs. We found weak invariance as the difference in ∆CFI (Model 4-Model 5, see Table 1) is less than |0.01| and RMSEA is less than 0.015 between models 4 and 5 (Chen 2007). That means constructs manifest in the same way within each group and that each item contributes to the latent construct to a similar degree in both groups.

We also tested differences in the structural paths between the two groups. We used a multi-group comparison to determine whether hypothesized relationships in our model differ between non-users and users. We tested for differences between these two groups by comparing an unconstrained structural model (model 10) with a constrained one (model 11) (‘constrained’ means that indicators’ loadings are held equal across the two groups). The constrained model showed a small decrease in fit indices, but still an acceptable fit. Nevertheless, the difference in goodness of fit between models 10 and 11 was significant (*χ*^*2*^ = 48.780, *p* ≤ 0.01). This finding implies significant differences in some of the structural paths of the two tested models. Thus, we proceeded to identify the paths that caused the difference in *χ*^*2*^. To do so, we constrained the regression paths, one at a time, and tested for differences in *χ*^*2*^ of each model to the baseline model. The results of the *χ*^*2*^ difference test per paths are depicted in Table 3.

#### Mediation Analysis

The mediation analysis showed that response efficacy influenced use indirectly through its effect on trust. As Table 3 depicts, respondents who are confident that the app enables effective response showed higher trust in the system. In turn, respondents who trust the system are more prone to use/continue to use the app. To test for the mediation of trust, we followed Hayes’s (2009) recommendations: (1) use bootstrapped sampling with at least 1,000 iterations (we did 2,000); and (2) consider the significance of the indirect paths as a sufficient condition for concluding the mediation is significant. To conduct this analysis, we needed a complete data set, for which we imputed 0.17 percent missing data using regression-based data imputation. The analysis showed that the standardized indirect effect was 0.078 (b = 0.128*0.613) for non-users and 0.106 for users (b = 0.192*0.551), and that the indirect effects were significant. The bias-corrected 95% confidence intervals for both non-users and users did not straddle zero. Thus, we concluded the mediation of trust is significant.

## Results

Table 3 is an overview of the results of our analysis. Information quality trust (H1) has a strong, positive effect on intention to use for both non-users (b = 0.128, *p* ≤ 0.05) and users (b = 0.192, *p* ≤ 0.01). Moreover, we found no statistical difference among non-users and users when it comes to the effect size of information quality trust. That means information quality trust has an equally positive effect on intention to use for both groups. Thus, H1 is supported.

Social influence (H2) has a strong positive effect on intention to use for both non-users (b = 0.290, *p* ≤ 0.001) and users alike (b = 0.280, *p* ≤ 0.001). As in H1, we found no statistical difference in effect size among the two groups. Thus, H2 is supported. In both groups, social influence has the strongest direct positive effect on protection motivation, which means friends, family, and members of an individual’s social network significantly influence the intention to use a warning app.

H3, which examined the positive effect of perceived vulnerability on protection motivation for non-users and users alike, was positive and significant only for the non-users group (b = 0.209, *p* ≤ 0.001). Hence, H3 is not supported.

H4, which hypothesized a positive effect of perceived severity on protection motivation in the two groups, was neither supported for non-users nor for users. That means we have no evidence to conclude that the belief that an emergency is going to be more disruptive will increase individuals’ intention to use the app or continue to use the app.

H5 tested whether maladaptive rewards have a stronger negative effect on protection motivation for non-users than for users. The effect sizes were significant for non-users (b = − 0.145, *p* ≤ 0.01) and user (b = − 0.249, p ≤ 0.001). The difference in the effect sizes of maladaptive rewards between non-users and users, however, was not statistically different, meaning that maladaptive rewards affects use intention similarly negatively in both groups. Thus, H5 is not supported.

H6a tested the positive effect of perceived response efficacy on protection motivation for non-users and users alike. For both groups, we tested the hypothesis considering the total effect of response efficacy on protection motivation, which equals the sum of direct and indirect effect of response efficacy. For non-users, the sum of the indirect (b = 0.078, *p* ≤ 0.05) and the direct effect (b = 0.280, *p* ≤ 0.001) showed that response efficacy has the strongest total positive effect on protection motivation for non-users (b = 0.358, *p* ≤ 0.001). Likewise for users, the sum of the indirect (b = 0.106, *p* ≤ 0.05) and direct effect (b = 0.183, *p* ≤ 0.1) showed that response efficacy has the strongest total positive effect on protection motivation (b = 0.288, *p* ≤ 0.05). Furthermore, we identified no significant difference in effect sizes between the two groups. Hence, non-users and users who perceive the app to be effective are similarly more prone to use it. H6b, the effect of perceived response efficacy on trust, was significant and positive for non-users (b = 0.613, *p* ≤ 0.001) and users (b = 0.551, *p* ≤ 0.001). We detected no differences between the groups in the multi-group analysis, thus supporting H6b.

H7 examined whether the negative effect of response cost on protection motivation is stronger for non-users than for users. Response costs are negative and significant only for users (b = − 0.155, *p* ≤ 0.05). Hence, H7 was not supported.

H8 tested whether self-efficacy has a stronger positive effect on protection motivation for users than non-users. Contrary to our hypothesis, we found that self-efficacy is negative and significant only for users (b = − 0.283, *p* ≤ 0.001). Thus, H8 was not supported.

Our results demonstrate that emergency experience has a significant, negative effect on users (b = − 0.131, *p* ≤ 0.05); at the same time, the effect of emergency experience is also significantly different between non-users and users.

## Theoretical Implications and Future Research

Our results provide empirical support for retaining the model we proposed to explain intention to use and to continue to use warning systems. Drawing on PMT and research on emergency systems, we conceptualized both use intention and continued use intention as manifestations of protective behavior motivation. We explained use intention and continued use intention incorporating determinants proposed by PMT, a theory originally developed to explain protective behavior in health and social psychology and subsequently applied successfully to explain the use of protective information systems (e.g., anti-malware software). Moreover, we added to PMT determinants from the emergency warning context which explain intention to carry out behaviors that are intrinsically protective, such as compliance with authorities’ recommendations. Overall, the analysis shows that integrating PMT with the determinants of protective behavior from research in emergency warning systems (i.e., trust and social influence) explains intention to use and continue to use more fully.

At the same time, our empirical analysis shows that our model explains intention to use (R2 = 69%) better than it explains continued use intention (R2 = 45%). In other words, the model explains better why non-users’ intend to begin using a warning system; it explains less well why current users intend to continue using a system. That suggests intention to continue using a system will be explained – at least in part – by different drivers than non-users’ intention to begin using a system. Users’ intention to continue to use the system entails more than motivation to protect oneself. For example, prior research on continued IS security use intention showed that users’ perceptions change over time as they gain firsthand experience (Vedadi and Warkentin 2020). In our research context, that would suggest that warning app users become progressively more aware of an app’s functions once they begin using it, and might experience disappointment because of limited functionalities; eventually, they may become less willing to continue using the app. In addition, determinants related to the degree to which warnings received via an app fit with the expectations and current needs of the user could influence continued use intention (Carter and Bélanger 2005). Such expectations could include how a user prefers to receive warnings and how helpful a user finds the warning messages to be, such as in terms of completeness of the information (Fischer-Preßler et al. 2020). Future research on warning apps, such as Covid-19 apps, could focus on user perceptions on the helpfulness of warning messages on mobile-enabled warn systems to gain a more complete picture of continued use intention in this context.

Other explanations about differences between non-users and users pertain to the nature of the emergency context. For instance, perceived *vulnerability* increases non-users’ intention to protect themselves, that is, to begin using the warning system. This is in accord with prior findings in campus emergency (Han et al. 2015) and with PMT postulations. However, vulnerability is irrelevant to motivate users to continue to use a mobile warning system – which is rather counterintuitive. In fact, we expected that non-users and users alike would be motivated to protect themselves using the warning app because they feel vulnerable. However, since vulnerability is only significant for non-users, vulnerability may become an insignificant driver for users as they begin to realize that warning messages are mostly about minor emergencies that do not pose a severe or acute threat to their personal safety. In Germany, our research context, major crises are relatively rare and cause few causalities, and major disasters such as floods, when they do occur, are typically confined to specific regions. That also means a PMT-based perspective may be better suited for warning systems that communicate high-impact (e.g., active shooters on U.S. campuses, hurricanes, wildfires) or more acute threats (e.g., Covid-19). This conclusion is aligned with the insignificant effect of perceived severity on both app use and continued use intention. Future research could test how the experience of acute or long-lasting emergencies such as epidemics influences use of mobile-enabled warning technology. For instance, with respect to Covid-19 tracing apps, how does having an infected friend or family member affect app use?

The non-significance of perceived severity also contradicts prior tests of PMT in IS security research to predict use (Boss et al. 2015) and continued use intention (Warkentin et al. 2016). However, unlike in our research context, studies in IS security applied strong fear-appeal manipulations to validate PMT. For instance, study participants were primed with a risk communication message about a would-be threat to their enterprise systems, such as the possibility of data loss under a set of conditions aimed at changing user behavior (Boss et al. 2015). In our survey, we opted not to do that. Rather than presenting the participants with a fictional experimental scenario, we controlled for personal emergency experience that, in prior research, was considered to influence the perception of a threat (Bubeck et al. 2012; Crossler et al. 2013) and constitutes an information source that can initiate protection motivation (Milne et al. 2000). This is also in line with the original theory’s scope of explaining long-term processes rather than reflexive responses (Rogers 1983). However, the control variable *crisis experience* did not have a positive effect on use and continued use intention, which could mean that perceived threats from emergencies decay over time and that PMT holds its explanatory power only in the context of acute or disruptive threats. In fact, the effect on continued use intention is negative, suggesting that experience with an emergency makes users less willing to continue using the app. Users who had a poor experience with an app during an emergency may consider that warning app useless, and, therefore, discontinue its use.

In our model, information quality trust is a mediator, not an exogenous variable as in Han et al. (2015). The theoretical reason for modeling information quality trust as such is that when trust refers to actionability and the relevance of the warning content, it has to be a function of what the user expects the system to fulfill in the first place. Thus, in our model, information quality trust is preceded by response efficacy. Our results differ from Han et al. (2015), who found that information quality trust has the strongest effect on intention. However, had we not modeled trust as a mediator, we would have underestimated the effect of response efficacy. Future research could seek to corroborate our result and test whether the (mediated) effect of response efficacy that showed significant in predicting protection motivation holds significance when the outcome variable is compliance intention.

The significant effect of response costs on users only suggests that non-users may initially disregard response costs, probably because to begin using the app does not come at the “cost” of major effort beyond installation and initial settings. However, when non-users become users, they consider costs important in deciding whether to continue using the app. Hence, app users should not experience using the app as time consuming or associate it with much effort.

Finally, our result on self-efficacy is somewhat surprising, as it seems to suggest that users who believe they can use an app effectively are less prone to continue using it. We refrain from this interpretation, however, since there is no theoretical justification for a negative causal relationship. One way to rationalize the reversed sign of self-efficacy is the effect of an omitted variable in our model. For instance, in research on SMS-based e-government services adoption, self-efficacy was found to have a positive effect on ease of use only indirectly through perceived behavioral control (Susanto and Goodwin 2013). Another possible explanation is that when a user’s level of self-efficacy grows, her expectations regarding the features of the app may increase as well. In other words, higher self-efficacy fosters discontinued app use because users realize they *could* protect themselves using the app and thus develop expectations about new features that may support counteraction even better – but have yet to be implemented. However, if the app fails to implement expected features, users realize that the app remains limited. When instead self-efficacy decreases, users are less interested in advanced features, which paradoxically may lead to a sense of satisfaction with the current version of the app and therefore foster continued use intention.

## Practical Implications

Our research model provides public officials with a framework for developing strategies to increase the adoption of warning apps. The positive impact of social influence calls for campaigns that promote using warning apps as an act of social responsibility. Another option is to integrate features such as “recommend this app to a friend” to leverage peer pressure. The positive effect of response efficacy, mediated by trust, suggests that it is critical that authorities build trust around them, which in turn increases people’s willingness to use warning apps. Conversely, public exposure of warning apps’ failures can decrease trust, such as the failure of a German warning app during a test in September 2020 (Zeit online 2020). These failures negatively influence perceived response efficacy, which is a major driver authorities can leverage to convince non-users to install an app. Furthermore, maladaptive rewards were significant for both user groups, which suggests that warning apps should be lean and use little memory and battery power. To promote a warning app, officials should emphasize the modest amount of system resources required to run the app.

For non-users, perceived response efficacy has a higher positive effect on intention to use than any of the dimensions of threat appraisal (perceived severity, vulnerability). That means it is imperative to maintain nationwide standards for public warning via an app, that is, warnings must be trustworthy and timely so as not to jeopardize the credibility of the app and confidence in its efficacy. In contrast, the more people know about governments’ efforts to provide accurate information, the more they trust them as a source. Another way to foster warning app use among non-users is to leverage perceived vulnerability, such as by illustrating how emergencies can affect people personally. When people perceive that they live in safe areas, warning systems look unnecessary. Thus, authorities should be specific in identifying plausible emergency events and explaining how an app helps protect against them. Finally, the reversed effect of self-efficacy in the users’ group suggests that once people start using the app, they begin judging its functionalities. As they become familiar with the app, issues surface and they demand new features. Just as with other apps, warning apps require continuous investments of resources, and authorities must make evident to users that an app is undergoing continual improvement.

## Conclusion

Our research develops and validates a model to explain what motivates use of mobile-enabled warning systems. We do so by integrating in our model the drivers identified by PMT scholarship as well as by IS research in emergency management. We compared the drivers to two groups, non-users and users. Analyzing group differences enables understanding whether drivers could change once individuals begin using the app. Users consider response cost, self-efficacy, and crisis experiences as relevant, while non-users do not. In contrast, non-users consider perceived vulnerability, which users overlook. For both, non-users and users, the model shows that trust, social influence, and response efficacy positively and maladaptive rewards negatively affect the intention to use and intention to continue use warning apps. We deem these findings particularly relevant to orient strategies that promote the use of warning apps. The increasing number of human-made and natural disasters worldwide has made mobile-enabled emergency communication a crucial asset for alerting the population. The intent of this research is also to provide practitioners with a theoretical understanding that will help them promoting emergency systems use and thus increase resilience of the population.

## Supplementary Information

Below is the link to the electronic supplementary material.

Supplementary file 1 (PDF 706 kb)
