# Supplementary material for: A Protection-Motivation Perspective to Explain Intention to Use and Continue to Use Mobile Warning Systems
Source: Bus Inf Syst Eng. 2021 Jun 16;64(2):167–82. doi: 10.1007/s12599-021-00704-0 (PMC8207817; doi:10.1007/s12599-021-00704-0)
Supplement: Supplementary file 1 — (PDF 706 kb) [file 12599_2021_704_MOESM1_ESM.pdf]

A Protection-Motivation Perspective to Explain Intention to Use and Continue to Use Mobile Warning Systems

Diana Fischer-Preßler, Dario Bonaretti, Kai Fischbach

Business & Information Systems Engineering (2021)

Appendix (available online via http://link.springer.com)

Appendix

Table B1 Constructs, definitions and references

Variable	Definition	Adapted from
Protection motivation	A non-user's initial intention of using a warning app.<br>A user's intention of long-term IS use over a period that covers estimates of future use.	Boss et al. 2015
Information quality trust	An individual's perception about the relevance of the information transmitted via the app.	Han et al. 2015
Social influence	An individual's perceived behavioral expectations of relevant others such as family, friends, or supervisors to use a warning app.	Han et al. 2015
Perceived vulnerability	An individual's estimate of how harmful the consequences of an emergency would be to things the individual values, which, in the context of this study, are personal health and security.	Han et al. 2015; Boss et al. 2015
Perceived severity	The degree an individual expects to be exposed to an emergency event.	Boss et al. 2015
Maladaptive rewards	The rewards associated with not using a warning app, such as capacity or battery savings on a smartphone.	Boss et al. 2015
Perceived response efficacy	The belief that the adaptive response (i.e., use of a warning app) will be effective in protecting someone.	Boss et al. 2015
Response costs	Costs associated with taking some action, which include time and effort to carry out a safety behavior.	Boss et al. 2015
Self-efficacy	An individual's level of confidence in her ability to use a warning app to protect oneself.	Boss et al. 2015
User status	An individual uses/not uses the app at present (user/non- user).	
Controls	Gender, Education, Age, Emergency experience: An individual's past experience with an emergency event.	Han et al. 2015

Table B2 Measurement items for the study

Construct	Item text	Adapted from reference
Perceived severity	If I were directly affected by a crisis, it would be a severe danger for me.<br>If I were directly affected by a crisis, it would pose a serious security risk to me.<br>If I were directly affected by a crisis, it would be a significant danger for me.	Johnston and Warkentin 2010
Perceived vulnerability	It is likely that a crisis could affect my safety in the future.<br>It is likely that a crisis could affect my health.<br>It is likely that a crisis could affect my security.<br>It is likely that there might be a crisis in the future.	Ada et al. 2016; Boss et al. 2015
Maladaptive rewards	Not using a warning app for crisis notifications and recommendations for action could save storage space on my smartphone.<br>Not using a warning app for crisis notifications and recommendations for action could save battery charge on my smartphone.<br>If I am not using a warning app for crisis notifications and recommendations for action, my smartphone would be less working to capacity.	Boss et al. 2015; Myyry et al. 2009
Response efficacy	An app for crisis notifications and recommendations for action increases the chance that I receive a crisis warning in a timely manner.<br>A warning app for crisis notifications and recommendations for action is effective in crisis situations.<br>A warning app for crisis notifications and recommendations helps warn me in a timely manner during crisis situations.	Johnston and Warkentin 2010; Mousavizadeh and Kim 2015

Self-efficacy	I'm sure I can easily use an app that warns me of crisis and gives me behavioral recommendations.<br>I am able to use an app that warns me of crisis and gives me behavioral recommendations without much mental effort.<br>It wouldn't be particularly difficult for me to interact with an app that warns me of crisis and gives me recommendations for behavior.	Lee and Larsen 2009; Boss et al. 2015
Social influence	Most people who are important to me would support me using an app that warns me of disasters and gives me behavioral recommendations.<br>Most people who are important to me think I should use an app that warns me about disasters and gives me behavioral recommendations.<br>Most people who are important to me would agree with my intention to use an app that warns me of disasters and gives me behavioral recommendations.	Bamberg et al. 2003
Trust	Using a warning app, I will be informed by this app only when facing a crisis.<br>I think I receive only relevant information using a warning app.<br>I do not think that I receive an excessive amount of information.	Han et al. 2015
Response costs	Using a warning app for crisis notifications and recommendations for action would be time consuming.<br>Using a warning app for crisis notifications and recommendations for action would require a considerable investment of effort.<br>Using a warning app for crisis notifications and recommendations for action decreases the convenience afforded by the application.	Boss et al. 2015
Protection motivation	I intend to use or continue to use a warning app for crisis notifications and recommendations for action.<br>I plan to use or continue to use a warning app for crisis notifications and recommendations for action.<br>I predict I will use or continue to use a warning app for crisis notifications and recommendations for action.	Boss 2015; Milne et al. 2002; Lee and Larsen 2009
Prior experience	Have you yourself ever been directly affected by a disaster as described above, so that your own health and/or safety was put at risk?	Developed for this study
Being a user	Are you using any of the following warning applications? Nina, Katwarn, or other? (yes/no)	

Table B3 Descriptives and Convergent validity

Construct	Indicator name	Mean	SD	Factor loading	Cronbach's alpha	Composite reliability	Average variance extracted (AVE)
Trust	Trust_3	5.191	1.561	0.818	0.901	0.905	0.647
	Trust_2	5.143	1.613	0.926			
	Trust_1	4.952	1.667	0.870			
Social influence	SNorm_3	5.259	1.497	0.925	0.918	0.923	0.694
	SNorm_2	4.704	1.778	0.845			
	SNorm_1	5.311	1.496	0.913			
Perceived vulnerability	PVul_4	4.002	1.699	0.896	0.954	0.955	0.741
	PVul_3	4.205	1.611	0.925			
	PVul_2	3.939	1.562	0.906			
	PVul_1	4.121	1.629	0.939			
Perceived severity	PSev_T_3	5.042	1.633	0.931	0.961	0.962	0.822
	PSev_T_2	5.051	1,594	0.991			
	PSev_T_1	5.257	1.504	0.915			
Maladaptive rewards	MalRew_3	2.746	1.642	0.867	0.905	0.905	0.646
	MalRew_2	3.018	1.741	0.898			
	MalRew_1	2.759	1.711	0.854			
Response efficacy	RespEff_3	5.61	1.486	0.940	0.945	0.946	0.758
	RespEff_2	5.599	1.5	0.909			
	RespEff_1	5.831	1.394	0.921			
Self-efficacy	SelfEff_3	6.254	1.112	0.864	0.905	0.906	0.646
	SelfEff_2	6.300	1.023	0.863			
	SelfEff_1	6.211	1.059	0.892			
Use intention	UI_3	5.092	2.033	0.969	0.978	0.978	0.885
	UI_2	5.02	2.07	0.969			
	UI_1	5.151	2.058	0.967			
Response cost	RCost_3	2.261	1.411	0.887	0.935	0.936	0.726
	RCost_2	1.993	1.31	0.924			
	RCost_1	2.224	1.428	0.920			

Table B4 Correlations and AVEs

Construct	1	2	3	4	5	6	7	8	9
1. Trust	*0.804*								
2. Social influence	0.547***	*0.833*							
3. Perceived vulnerability	0.382***	0.501***	*0.906*						
4. Perceived severity	0.425***	0.358***	0.436***	*0.861*					
5. Maladaptive rewards	-0.389***	-0.413***	-0.208***	-0.144*	*0.804*				
6. Response efficacy	0.615***	0.623***	0.489***	-0.369***	-0.523***	*0.871*			
7. Self-efficacy	0.391***	0.459***	0.255***	0.251***	-0.295***	0.467***	*0.804*		
8. Response cost	-0.546***	-0.601***	-0.380***	-0.290***	0.613***	-0.692***	-0.542***	*0.852*	
9. Use intention	0.537***	0.680***	0.553***	0.260***	-0.587***	0.717***	0.374***	-0.702***	*0.940*

The diagonal (italicized) represents the square root of the AVE scores.

p < 0.001; **p < 0.01; *p < 0.05

Table B5 Invariance tests

Type of invariance	Description	Retained	Implication
Configural	Interpretation: The same CFA model is specified for each group, but parameters are freely estimated in each group. The models separately shows good fit.<br>Operationalization: The measurement model of non-users and users fit well compared to the unconstrained simultaneous measurement model.	Yes. Model fit of Model 4, Model 2, & 3 are good.	The factor structure operates similarly between groups. Still, factors may have unequal pattern coefficients, intercepts, or error variances for some indicators.
Metric (or "weak")	Interpretation:<br>Equality constraints are imposed on unstandardized factor loadings.<br>Operationalization: The constrained (factor loadings) simultaneous measurement model fits well compared to the unconstrained simultaneous measurement model.	Yes. Difference in ΔCFI (Model5-Model4) < |.01| and RMSEA < .015 between model 4 and Model 5 (Chen 2007).	The constructs manifest in the same way within each group. Factors scores are calculated using the same weighting schemes in both groups because the slopes of regressing the indicators on their respective factors are equal.
